# Supplementary material for: Precision Phenotyping of Nectar-Related Traits Using X-ray Micro Computed Tomography
Source: Cells. 2022 Oct 31;11(21):3452. doi: 10.3390/cells11213452 (PMC9658521; doi:10.3390/cells11213452)
Supplement: Supplementary file 1 [file cells-11-03452-s001.zip › cells-1960424-supplementary.pdf]

♂

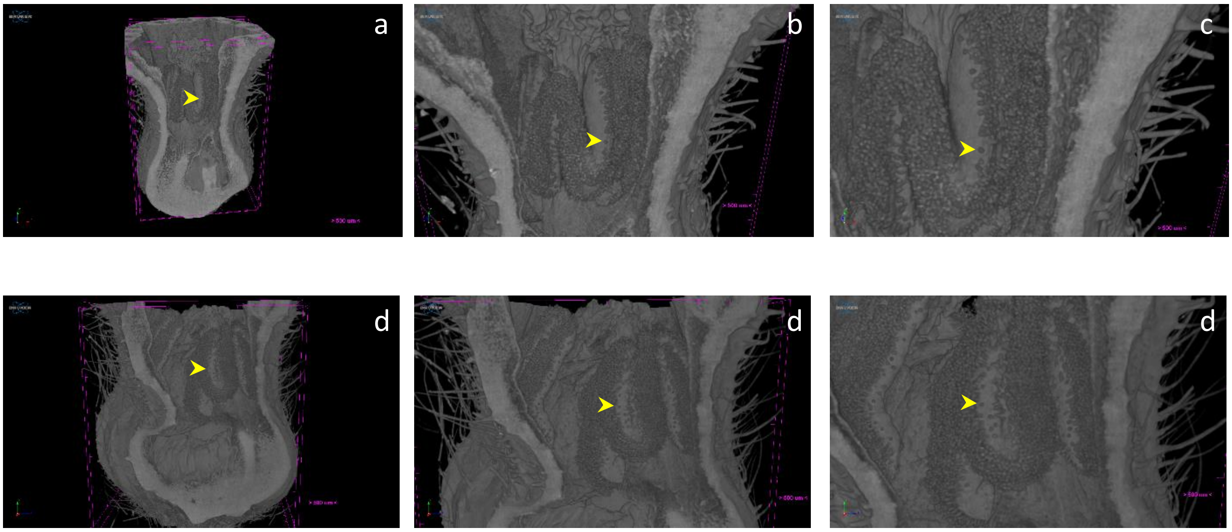

Figure S1. Pollen on Stamens in ♂ (a, b, c) and (d, e, f) flower types at different magnifications. Yellow arrows indicate same grains of pollen at different magnification. Male = ♂; hermaphrodite = . Scale of 500µM is specified inside each image.

Table S1. Nectar-related traits measured in male and female flowers.

Trait	Sex	Mean	Std Deviation	n
Flower width (mm)	♂	4.44	0.40	11
	♀	6.17	0.52	9
Nectary cross-section area (mm^2)	♂	2.55	0.62	11
	♀	2.83	1.16	9
Nectary surface area (mm^2)	♂	20.17	4.58	11
	♀	49.69	16.05	9
Nectary volume (mm^3)	♂	4.89	1.47	11
	♀	13.46	6.00	9

Table S2: Correlation analysis between nectary volume, nectary cross-section area, nectary surface, flower width and nectar volume in the respective male, female and pooled melon flowers.

♂	*Nectary volume*	*Nectary area*	*Nectary surface*	*Flower width*	*Nectar volume*
Nectary volume	1				
Nectary area	0,92213072	1			
Nectary surface	0,87459246	0,915253244	1		
Flower width	0,33781784	0,212381412	-0,039258879	1	
Nectar volume	0,84601965	0,821375837	0,748207848	0,392382802	1
♀	*Nectary volume*	*Nectary area*	*Nectary s urface*	*Flower width*	*Nectar volume*
Nectary volume	1				
Nectary area	0,99329736	1			
Nectary surface	0,98720845	0,98055743	1		
Flower width	0,46333634	0,476220668	0,393290503	1	
Nectar volume	0,6771372	0,749450083	0,639449907	0,430519311	1
♂♀	*Nectary volume*	*Nectary area*	*Nectary surface*	*Flower width*	*Nectar volume*
Nectary volume	1				
Nectary area	0,75345199	1			
Nectary surface	0,9839094	0,678285618	1		
Flower width	0,77665457	0,315486737	0,79489919	1	
Nectar volume	0,86011885	0,438456586	0,891058915	0,875490593	1

Table S3: Raw data used for the correlation analysis between nectar volume and nectary attributes e.g. nectary volume, nectary cross-section area, nectary surface and flower width in the respective male, female and pooled melon flowers.

Sample type	Sample number	Nectary volume (mm^3)	Nectary Area (mm^2)	Nectary Surface (mm^2)	Flower Width (mm)	Nectar volume (µl)
♀	F1	13,4501	3,067475	51,81187025	5,585	26,55172414
♀	F2	23,0517	4,674175	70,64769829	6,56	27,75862069
♀	F3	10,6201	2,4776875	41,38364725	6,61	22,4137931
♀	F4	23,2675	4,6512125	79,66625748	6,325	20,17241379
♀	F5	13,0232	2,7926625	45,78910043	6,97	19,31034483
♀	F6	13,1429	2,601375	49,00152689	6,235	16,03448276
♀	F7	8,9979	1,93015	38,64649759	5,73	12,4137931
♀	F8	9,91	2,140075	41,94929774	6,24	16,37931034
♀	F9	5,7	1,2181375	28,39522302	5,34	10
♂	M1	7,323	3,514925	24,76395872	5,195	3,67
♂	M2	3,5626	1,895075	15,35367116	4,89	2,93
♂	M3	2,9474	1,99535	13,62806135	4,32	1,92
♂	M4	2,7837	1,6563625	12,82838728	4,355	1,22
♂	M5	6,9442	3,40015	23,86520414	4,795	3,45
♂	M6	4,6542	2,6569625	21,10191387	4,425	2,59
♂	M7	6,0017	2,7890125	22,78087666	4,405	3,45
♂	M8	4,9983	2,519275	20,94963851	3,88	2,84
♂	M9	5,2221	3,217925	26,79056616	3,8605	2,93
♂	M10	5,0035	2,2345875	21,13782548	4,215	2,07
♂	M11	4,3929	2,2361875	18,72263669	4,545	2,27

Table S4. Nectar volume measurements in male and female flowers.

Nectar volume sample	Sampling type	Mean (µl)	Std deviation	Number of samples
M1	Intact	2,18	0,48	11
M2	Dissected	2,21	0,71	11
F1	Intact	8,83	3,17	9
F2	Dissected	19,61	5,17	9
